# Effect of electro-acupuncture on ovarian function of women with diminished ovarian reserve: study protocol for a randomized controlled trial

**DOI:** 10.1186/s13063-021-05894-2

**Published:** 2021-12-14

**Authors:** Le Yang, Hanwang Zhang, Li Zhou, Ying Gao, Lijuan Yang, Yajun Hu, Lu Xu, Dongmei Huang

**Affiliations:** 1 Tongji Hospital, Tongji Medical College, Huazhong University of Science and Technology, 1095 Jiefang Avenue, Wuhan, 430030 Hubei China; 2 First Hospital of Wuhan, 215 Zhongshan Avenue, Wuhan, Hubei China; 3 Union Hospital, 1277 Jiefang Avenue, Wuhan, Hubei China; 4 Reproductive Hospital, Jiangxi University of Traditional Chinese Medicine, 597 Shuangmashi Road, Nanchang, Jiangxi China

**Keywords:** Electro-acupuncture, Diminished ovarian reserve, In vitro fertilization-embryo transfer, Randomized controlled trial, Study protocol

## Abstract

**Background:**

Diminished ovarian reserve (DOR) is the precursor state of ovarian failure and can cause the decline of women’s reproductive function. DOR also leads to poor outcome of in vitro fertilization and embryo transfer (IVF-ET) by affecting the oocytes, high qualified embryo rate, pregnancy rate, etc. Some studies have demonstrated that acupuncture can improve ovarian function. But to date, there is limited evidence indicating that acupuncture or electro-acupuncture is efficient to DOR. This trial aims to evaluate the efficiency and safety of electro-acupuncture for the ovarian function and the following outcome of IVF-ET in DOR patients.

**Methods:**

This will be a multicenter randomized controlled clinical trial. A total of more than 338 women with DOR will be randomly allocated to treatment and control groups in 1:1 ratio receiving acupuncture before undergoing IVF-ET. The primary outcome will be the clinical pregnancy rate per cycle of IVF-ET after acupuncture. The secondary outcomes will be ovarian reserve function, outcomes of IVT-ET, blood biochemical index, Massachusetts General Hospital Acupuncture Sensation Scale (MASS), scores from the self-rating anxiety and depression scale, quality of life, and pregnancy outcomes. The safety of acupuncture will also be assessed.

**Discussion:**

The results of this trial may provide high-quality evidence regarding the effectiveness of electro-acupuncture in the treatment of DOR and following outcomes of IVF-ET. This will also help patients with DOR and their physicians by offering a new treatment option.

**Trial registration:**

Chinese Clinical Trial Registry ChiCTR1900024626. Registered on 19 July 2019.

**Supplementary Information:**

The online version contains supplementary material available at 10.1186/s13063-021-05894-2.

## Background

Diminished ovarian reserve (DOR) indicates a reduced number of retrieved follicles and the reduction of the quality of oocytes affected by non-biological reasons before the age of 40. Multiple studies consider that the cause of DOR is related to age, genetic factors, immune factors, social and environmental factors, infection, iatrogenic injuries, multiple pregnancies, etc. Normally, the ovarian reserve function declines at the age of 30, and fall off sharp after 35. The incidence rate of DOR is climbing and shows a younger trend. However, there is no effective pharmacotherapy at present.

In-vitro fertilization-embryo transfer (IVF-ET) is a main therapy treating infertility, and controlled ovarian hyperstimulation (COH) is the key step. In IVF-ET, the success of COH depends on the ovarian reserve function and ovarian response. A clinical trial finds that when undergoing IVF-ET, the DOR patients’ initial dose of gonadotropin (Gn), total Gn, Gn days, and abortion rate are obviously higher than patients with normal ovarian function, but the oocytes, M II oocytes rate, high qualified embryo rate, fertilization rate, implantation rate, and pregnancy rate are all distinct inferior to patients with normal ovarian function [1]. In addition, DOR can cause reproductive endocrine dysfunction including less menstrual volume, menstrual loss, and amenorrhea, which further affects women’s reproductive function. Besides, it also leads to perimenopause-related symptoms, such as hot flashes, night sweats, irritability, etc. All these symptoms impact on patients’ quality of life. And it will develop into premature ovarian failure (POF) within 1–6 years when without intervention at an early stage. Hence, a new therapy for DOR is necessarily required.

In recent years, as an important part of traditional Chinese medicine, acupuncture has attracted tons of attention in the fields of reproductive endocrine and infertility worldwide [2–4]. Acupuncture can stimulate the neuro-endocrine system, regulate the overall endocrine level and ovarian microenvironment as a whole, improve the internal environment of follicles, improve ovarian hemodynamics, and thereby promote follicular development and ovulation. In addition, acupuncture can also enhance endometrial receptivity and relieve patients' tension and anxiety in assisted reproductive technology (ART) [5]. Acupuncture, which can improve ovarian function, is now widely used in the treatment of POF. Two Meta-analysis show that acupuncture is a relative effective intervention treating POF, which helps for recovering menstruation and boosting level of serum follicle-stimulating hormone (FSH) and estradiol (E2). The curative is sustainable till a month after the end of treatment [6, 7]. Since DOR can develop to POF within 1–6 months, why do not we intervene it in the early DOR phase in order to delay or reverse the progress to POF?

Given its effect on ovarian function, acupuncture is expected to be a new option for the treatment of DOR. But to date, the available evidence of the efficacy of acupuncture or electro-acupuncture for DOR remains insufficient. Only a few small sample, non-randomized controlled clinical trials suggest that acupuncture or electro-acupuncture can improve ovarian reserve function of DOR patients [8, 9]. Therefore, a large sample, multicenter, randomized controlled trial is designed to evaluate the efficiency and safety of electro-acupuncture in DOR patients. The clinical pregnancy rate is the primary outcome reflecting ovarian function. The following outcome of IVF-ET will also be evaluated as a secondary outcome.

### Objectives

Through this study, we expect to test the following hypotheses: (1) evaluate whether electro-acupuncture is superior to the control group in ameliorating ovarian function of DOR patients reflected by improving clinical pregnancy rate, and (2) assess the safety of electro-acupuncture.

## Methods

### Trial design

This will be a multicenter, randomized, controlled clinical trial. The trial was designed as a superiority trial, with the aim of evaluating whether the electro-acupuncture intervention was superior to the control group in improving ovarian function of DOR patients. A total of more than 338 women with DOR will be randomly allocated to treatment and control groups in 1:1 ratio receiving acupuncture before undergoing IVF-ET.

**Fig. 1:**
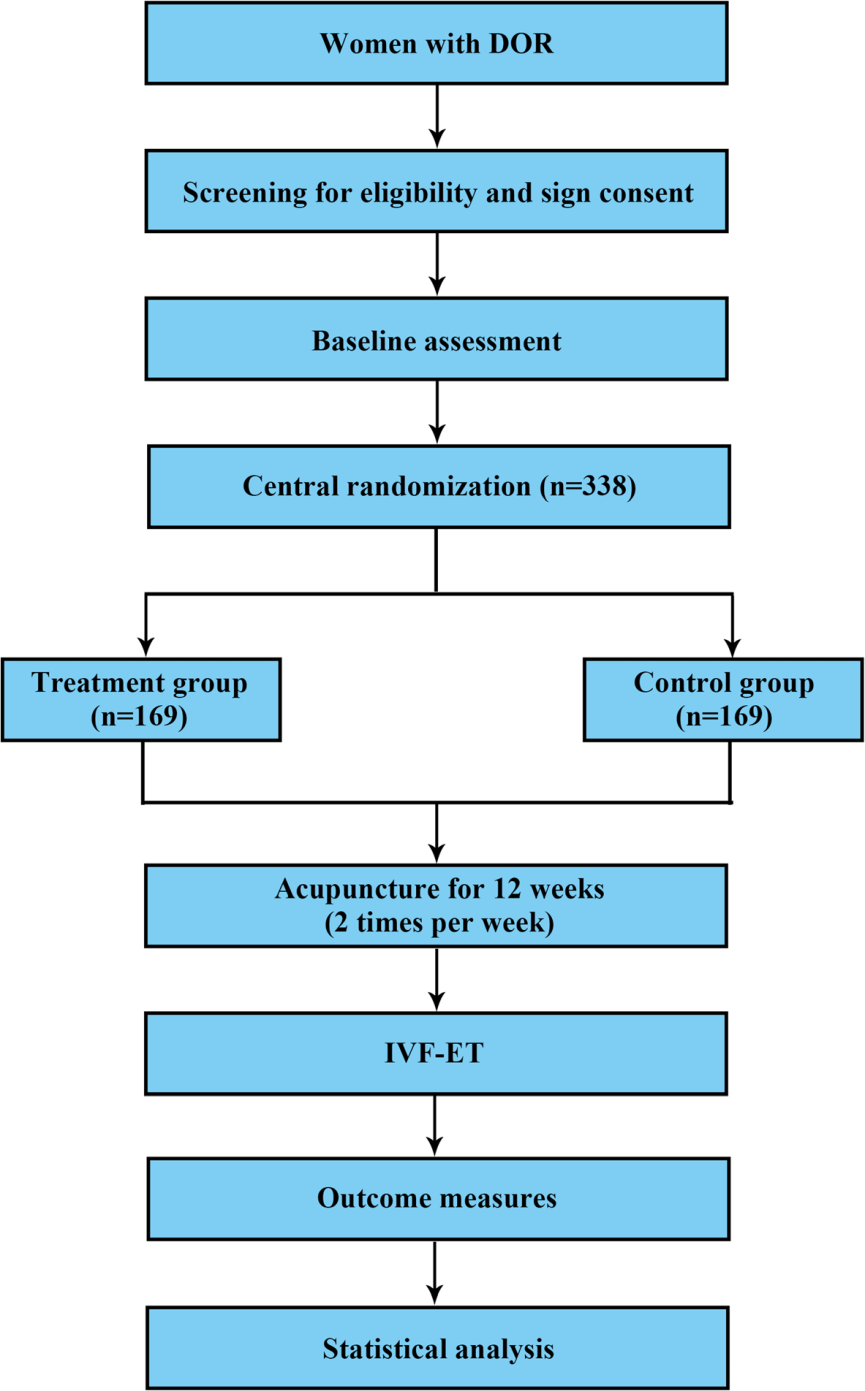
Flowchart of the trial procedures

This trial has been registered at the Chinese Clinical Trial Registry (ChiCTR1900024626). The flowchart and study design schedule are presented in Fig. 1 and Table 1. The Standard Protocol Items: Recommendations for Interventional Trials (SPIRIT) checklist is provided as Additional file 1.

**Table 1:**
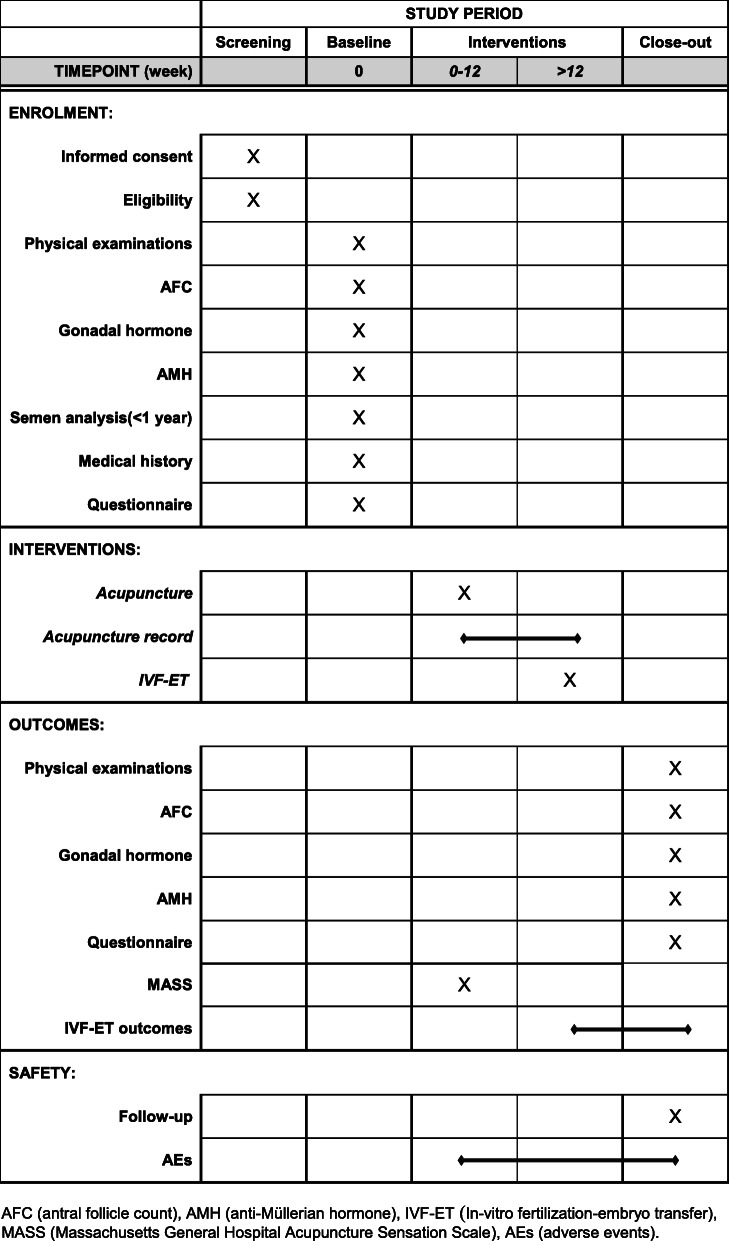
Participants timeline

*AFC* antral follicle count, *AMH* anti-Müllerian hormone, *IVF-ET* In-vitro fertilization-embryo transfer, *MASS* Massachusetts General Hospital Acupuncture Sensation Scale, *AEs* adverse events

### Study setting

Patients will be recruited from the following 4 hospitals in China: Tongji Hospital affiliated to Tongji Medical College of Huazhong University of Science and Technology, Union Hospital affiliated to Tongji Medical College of Huazhong University of Science and Technology, Wuhan First Hospital, and Reproductive Hospital affiliated to Jiangxi University of Traditional Chinese Medicine.

### Consent to participate

This protocol is in accordance with the principles of the Declaration of Helsinki. Written informed consent will be obtained from patients prior to enrolment. The informed consent is provided as Additional file 2. In each sub-center, two independent gynecologists are responsible for recruiting patients and gaining informed consent. On the consent form, participants will be asked if they agree to the use of their data when they choose to withdraw from the trial. Participants will also be asked for permission for the research team to share relevant data with researchers from the universities taking part in the research or from regulatory authorities, where relevant. This trial does not involve collecting biological specimens for storage. No changes are made to the trial protocol after the subject recruitment started.

### Inclusion criteria

Participants will be included if they meet the following criteria: (1) age < 40, will undergo IVF-ET; (2) low ovarian reserve: antral follicle count (AFC) < 7, or AMH< 1.1 ng/ml; or has a history of poor ovarian response: in the last hyperstimulation cycle, the number of retrieved oocytes < 3; (3) spouse’ semen examination is normal, or after semen prewash can reach the standard of common IVF or intracytoplasmic sperm injection (ICSI).

### Exclusion criteria

Participants will be excluded if they meet any of the following criteria: (1) male with azoospermia; (2) male/female’s chromosome is abnormal; (3) adenomyosis, uterine fibroids, endometrial polyps, scar uterine, reproductive system tuberculosis, oviduct effusion, pelvic lesions such as ovarian endometriosis cyst or tumor; (4) female has other endocrine diseases: thyroid diseases, hyperprolactinemia, insulin resistance, diabetes, adrenal diseases, etc.; (5) definitively diagnosed autoimmune diseases such as systemic lupus erythematosus, rheumatoid arthritis, and antiphospholipid syndrome; (6) other pathogenesis that leads to recurrent miscarriage or agnogenic recurrent miscarriage; (7) a history of cancer and has received radiotherapy and chemotherapy; (8) had acupuncture treatment in recent 3 months; and (9) unwilling to sign the informed consent of this study.

### Randomization and masking

Internet-based central randomization system will be applied in this trial. Three hundred thirty-eight participants will be randomly allocated to the treatment group and control group in a 1:1 ratio. The Internet-based central randomization system designs the random parameters and allocation. The appointed researchers in each hospital will apply for the group assignment allocated by the system through inputting a specific code. The randomization protocol and the parameters set during it are collectively called blinding code, which are kept strictly by researchers who are not involved in the process of outcome evaluation and statistical analysis.

### Interventions

To improve patients’ adherence to interventions, electro-acupuncture will be performed by acupuncturists with over 5 years of clinical acupuncture experience and obtained a license from the Ministry of Health of the People’s Republic of China. Before performing the treatment, acupuncturists will receive centralized training including an introduction to the basic clinical research methods and a practical demonstration of the treatment. WeChat will also be used for making appointments and regular reminders. At each part of the study, including assessing ovarian function before and after the intervention, performing electro-acupuncture, or making follow-up visits, the independent gynecologist will make appointments and remind participants of some notes by using this APP.

The treatments of both two groups start after a spontaneous period or a withdrawal bleeding by progestin. For patients with amenorrhea or oligomenorrhea, the withdrawal bleeding is induced using progesterone by the outpatient doctors, and then the intervention starts after the withdrawal bleeding. The acupuncture protocols are formulated according to the theory of traditional Chinese medicine. The number of needles used, methods of stimulation (manipulation and electro-acupuncture), frequency, and time of the treatment are detailed described following the CONSORT and STRICTA recommendations [10].

Fixed protocols are used for both the treatment group and the control group. All patients receive the treatment of active acupuncture or sham acupuncture, twice or three times a week with a maximum of 24 times in 8-12 weeks. Treatments will not be performed during menstruation in both two groups. The treatment date, time, the name of the acupuncturist and the intensity of the electro-acupuncture stimulation which can vary between the different electrodes are recorded when the patient receives acupuncture treatment. The range of the intensity of the electro-acupuncture stimulation will be recorded, e.g., 1.2–3.0 mA.

#### Treatment group:

For the treatment group, two groups of acupoints will be used alternatively.

The first group consists of governor vessel (GV) 20, conception vessel (CV) 6, CV 3, bilateral stomach (ST) 29, bilateral spleen (SP) 6, bilateral ST 36, and bilateral pericardium (PC) 4. The patients will be asked to stay in a supine position and keep the whole body relaxed and comfortable. Disposable sterilized needles (size: 0.25 × 40/50 mm) will be inserted into a depth of 15~35 mm and stimulated manually to evoke needle sensation (“Deqi” in traditional Chinese medicine, TCM). And then CV 3 and CV 6, bilateral ST29, and SP6 and SP 9 bilaterally will be thereafter connected to electrical stimulators (Export Abteilung, Schwa-Medico GmbH, Wetzlarer Str. 41-43; 35630 Ehringshausen) and stimulated with low-frequency of 2 Hz, 0.3 ms pulse length for 30 min. The intensity was adjusted to the maximum tolerated intensity of the patients. The other needles without electrical stimulators will be manually stimulated to evoke “Deqi” every 10 min.

The second group of acupoints consists of the bladder (BL) 23 and 32 bilaterally, bilateral kidney (KI) 3 and SP 6, and GV 20. Patients will be asked to stay in a prone position and keep the whole body relaxed and comfortable. GV 20 will be punctured obliquely, and other acupoints will be perpendicularly needled into a depth of 15~35 mm using disposable sterilized needles (size: 0.25 × 40/50 mm). All acupoints will be stimulated manually to evoke needle sensation (“Deqi” in TCM). And then BL 23 and 32, and KI 3 and SP 6 bilaterally will be stimulated with low-frequency in the same way as the first group. GV 20 will be stimulated manually to evoke “Deqi” every 10 min.

#### Control group

For the control group, four pseudo-acupoints are used, with two points on each shoulder and the two on each upper arm, which are not located on any meridians. The patients will be asked to stay in a supine position and keep the whole body relaxed and comfortable. Disposable sterilized needles (Size: 0.18 × 25 mm) will be inserted superficially to a depth of < 5 mm without any manual stimulus and the needle sensation (“Deqi” in TCM) should not be evoked. Electrodes are connected to the needles, but the stimulators should be turned on at an intensity of zero. Each intervention lasts for 30 min.

#### IVF-ET treatment

After the acupuncture treatment, patients’ ovarian reserve function will be evaluated again, and then IVF-ET cycle treatment will be conducted. This will be performed by the reproductive medicine center according to the patients’ situation and the standard procedure.

### Concomitant treatments

Drugs and other treatments, which may interface the evaluation of electro-acupuncture effect, will be discouraged. Discouraged treatments include sexual hormones, contraceptives, and herbs. If treatment not recommended in this trial has already been performed, relevant information should be recorded in the patient’s case report form.

### Outcome measures

#### Primary outcome measure

The primary outcome is clinical pregnancy rate per cycle of IVF-ET after acupuncture (through study completion, an average of 1 year). Clinical pregnancy was defined by the presence of a fetal heartbeat at 6–7 weeks of pregnancy. The clinical pregnancy rate is the number of clinical pregnancies per embryo transfer cycles.

#### Secondary outcome measures

##### The change of ovarian reserve function

Assessing patients’ ovarian reserve function before and after acupuncture intervention (at 0 weeks and up to 12 weeks), including:

1. The serum AMH, inhibin, and FSH and E2 levels on the third day of menstruation;
2. AFC.

##### Outcomes of IVF

Outcomes of IVF include:

1. Gn dosage and usage days;
2. E2 level and endometrial thickness on human chorionic gonadotropin (HCG) day;
3. Number of oocytes;
4. MII oocytes;
5. Normal fertility rate;
6. The number of available embryos;
7. Number of high-quality embryos;
8. Cycle cancelation rate (including cycle cancelation rate caused by various reasons);
9. Implantation rate: including fresh periodic implantation rate, per cycle implantation rate and cumulative implantation rate;
10. Fresh cycle clinical pregnancy rate and cycle cumulative clinical pregnancy rate;
11. Early, mid, and late pregnancy abortion rate;
12. Risk of ovarian hypertrophy and incidence of obstetric complications;
13. FSH, LH, E2, and AMH in follicular fluid; oxidative stress-related indicators such as reactive oxygen species (ROS), superoxide dismutase (SOD) level, etc.;
14. Live rate: including fresh cycle live rate, cycle live rate and cumulative live rate.

These outcomes will be assessed through study completion, at an average of 1 year.

##### Blood biochemical index examination before and after acupuncture

The level of blood corticotrophin-releasing hormone (CRH), norepinephrine, adrenaline, 5-hydroxytryptamine, beta-aminobutyric acid (GABA), dopamine (DA), and neuro-endorphin will be tested before and after treatment (at 0 weeks and up to 12 weeks).

##### Deqi sensation scale of acupuncture

After each acupuncture treatment, patients will be asked to rate the MASS [11] independently by evaluating 12 acupuncture sensation degrees within 5 min. The higher the weighted total score is, the more obvious is the Deqi sensation degree.

##### Questionnaires

Evaluation of anxiety, depression, and quality of life will be performed before and after treatment (at 0 weeks and up to 12 weeks), including:

1. Baker anxiety self-rating scale (BAI) and baker depression self-rating scale (BDI-II): higher score indicates higher degree of depression or anxiety [12, 13].
2. Zung anxiety self-rating scale (Zung-SAS), Zung depression self-rating scale (Zung-SDS) [14, 15].
3. Quality of life measurement (QOL): Quality of life will be assessed by SF-36 and the Chinese quality of life scale (CHQOL).

##### Follow-up detection

Pregnancy patients will be followed up to the end of pregnancy. Adverse pregnancy outcomes and live birth rates will be recorded. Non-pregnant patients will be followed up for 1 year after treatment, testing the ovarian reserve function, follow-up treatment, and pregnancy status of the patients within 1 year.

### Participant retention

All of the participants, regardless of which group, will receive health education. Health education includes: (1) patients will be encouraged to engage in an appropriate exercise training program, (2) patients will be suggested to continue striving for a healthy diet, and (3) patients will be given relaxing training and psychological counseling. For pregnant patients, 3D ultrasound will be appointed in advance and advices during pregnancy and perinatal will be given by gynecologists. Non-pregnant patients will also be followed up for 1 year after treatment and given advices about following treatment like ovulation-inducing scheme of IVF-ET.

### Adverse events

Adverse events (AEs) will be classified. AEs related with electro-acupuncture include bleeding after needle withdrawal, striking of needle, bending of needle, and fainting during acupuncture treatment. AEs related to IVF-ET include ovarian hyperstimulation syndrome, colporrhagia and infection caused by egg retrieval, and multiple pregnancy. Researchers will obtain AEs by inquiring of the participants at each treatment or follow-up visit or by voluntarily reporting by the participants. The percentage of AEs and severe adverse events that occurred during treatment will be recorded in detail. Chi-square test is used to analyze the total proportion of adverse events in each treatment regimen and the differences between the classifications. Unless otherwise formally requested, each report from the safety monitor board will report details and summaries of adverse events in a double-blind manner.

### Criteria for discontinuing interventions

Acupuncture will be ended if participants meet any of the following conditions: (1) serious adverse events occurred during or after treatment, (2) misdiagnosed after randomization, and (3) patients found to be pregnant after randomization.

### Provisions for post-trial care

There is no anticipated harm and compensation for trial participation.

### Sample size

According to studies conducted by Hong SB et al. and Roustan A et al., the clinical pregnancy rate of DOR patients is respectively 20.6% and 20.2% [16, 17]. Due to the lack of effective randomized blinded controlled clinical trials that choose clinical pregnancy rate per cycle as the primary outcome measure in evaluating acupuncture efficacy, we presume 15% for the patient's clinical pregnancy rate per cycle. Assuming that the clinical pregnancy rate can be increased by 15% after acupuncture treatment, *α*=0.05, *β*=0.20, power is 80%. The sample size is calculated according to the following formula [18], nA=κnB, nB=[pA(1-pA)/κ+pB(1- pB)](z1-α/2+z1-β)2/(pA-pB)2,*κ*=1, then we get nA=nB=135,which means the sample size of each group is 135. Allowing for a 20% dropout rate, the sample size of this trial is 338 participants.

### Recruitment

Eligible patients will be recruited from participating hospitals through the following strategies. Posters will be placed on doctors’ offices, bulletin boards, or other places in the hospitals. Advertisements are also put via network, Wechat, etc. Eligible patients from the outpatient and inpatient clinics will be advised by doctors. In each hospital, specialized staff will contact patients and make an appointment for a screening visit.

### Blinding

In this study, participants, outcome assessors, and statisticians are blinded. The acupuncturists will not be blinded.

Many efforts will be done to maintain blinding in participants assigned to the intervention and control groups. For example, eligible patients will be acupuncture naïve. In the control group, electrodes are connected to the needles, which is the same as the intervention group, but the stimulators should be turned on at an intensity of zero.

We will also try to ensure that the outcome assessors and statisticians are unaware of the group allocation. The two groups will be labeled as groups 1 and 2. Assessors will be randomly assigned to follow-up. Allocation sequence will be concealed until the end of the study. In addition, we will separately give relevant data of AEs, and AES to an independent statistician for analysis, while the statistician responsible for the analysis of primary outcome and other secondary outcomes will be blinded to group allocation.

After the patients completed the whole visit, they will be told which group they were allocated in.

### Data management and quality control

Researchers including acupuncturists, outcome assessors, and statisticians will receive training about data management. In this trial, online monitoring will be used. Data of participants will be inputted into the electronic case report form (eCRF) through clinical trial management public platform called ResMan. The clinical research associates are responsible for verifying the accuracy of data. All the research documents, which include both the paper files and electronic documents, will be preserved for at least five years after publication. The private information of patients (name, telephone number, and ID number) will be anonymous to ensure participant confidentiality. After approval of a proposal and with a signed data access agreement, researchers whose proposed use of the data has been approved can have access to the data.

The quality controller of each hospital will complete self-inspection at least once every month. Remote or onsite monitoring will be performed for all centers once per 3 months by the principle investigator. The auditing will be done by DMC (Clinical Research Center of Tongji hospital) at the beginning, middle, and end of the trial. The DMC was established in 2018, which consists of investigators, data managers, biostatisticians, and study coordinators. The DMC is independent of the study organizers. The responsibility of DMS is periodically reviewing the accumulating data and determining if this trial should be modified or discontinued. The DMC does not have executive power; rather, it communicates the outcome of its deliberations to the trial sponsors. The chair is Mr. Zhou JF, with Dr. Xu SB, Dr. Zhu XJ. For patients who deviate from the trial, causes and relevant outcome data will be recorded in a case report form as much as possible.

### Statistical methods

All analyses will be based on the intention-to-treat principle. Data analysis will be performed by an independent statistician using SAS statistical software (version 9.4; SAS Institute Inc.).

The analysis of variance (ANOVA) will be used to test the difference of clinical pregnancy rate (primary outcome) between the two intervention groups with the baseline date as a variate and taking into account the multicenter effect. Missing data will be imputed using the multiple imputation method. Continuous data will be presented as the mean and standard deviation, or the median and interquartile range. Categorical data will be presented as the number and percentage. Comparisons between groups will be analyzed using an independent *t* test or Wilcoxon rank-sum test for continuous variables, and a chi-square test or Fisher exact test for categorical variables. All statistical tests will be two-sided, and *p* < 0.05 will be considered statistically significant.

## Discussion

This trial aims to determine whether electro-acupuncture can improve ovarian function and clinical pregnancy rate during IVF cycles for DOR patients. Some clinical and experimental studies have found that acupuncture can promote the follicular development and ovulation, and further improve ovarian function [6–9]. A few clinical trials have shown effectiveness in treating DOR through acupuncture or electro-acupuncture [8, 9, 19, 20]. The pregnancy rate in IVF-ET after acupuncture is assessed by several studies [21, 22]. However, the population of these trials are all small sample and non-controlled. Therefore, we will confirm the efficacy by a large sample, multi-center randomized controlled clinical trial. This trial will assess the clinical pregnancy rate as the primary outcome reflecting ovarian function.

In this trial, the form of the electro-acupuncture is manual acupuncture. All acupoints will be stimulated manually to evoke a needle sensation called Deqi. After that, the electrodes will be connected to the needles and start simulating. The intensity of Deqi sensation is critical for the effects of acupuncture [23]. The MASS will be used to quantify the intensity of Deqi sensations after each treatment, which aims to explore the association between the intensity of Deqi sensations and the efficacy of electro-acupuncture.

There are some limitations in this trial. The acupuncture protocol consists of two groups of acupoints, which seems to be a little sophisticated for acupuncturists, and may increase the difficulty in implementing placebo acupuncture. Besides, the acupuncturists in this trial will not be blinded to the intervention. The solution of the situation is blinding the group assignment before electro-acupuncture.

In conclusion, the results of this trial may provide high-quality evidence evaluating the effectiveness of electro-acupuncture in the treatment of DOR patients and following outcomes of IVF-ET. This study will contribute to offer a therapy option for DOR patients.

## Trial status

This trial is currently recruiting participants. The protocol version number and date: V3.0, September 8, 2020. The recruitment began on August 1, 2019. The estimated completion date of recruitment is August 1, 2023.

## Supplementary Information

**Additional file 1:.** SPIRIT checklist

**Additional file 2:.** Informed consent

## Abbreviations

DOR: Diminished ovarian reserve
IVF-ET: In vitro fertilization and embryo transfer
MASS: Massachusetts General Hospital Acupuncture Sensation Scale
COH: Controlled ovarian hyperstimulation
Gn: Gonadotropin
POF: Premature ovarian failure
ART: Assisted reproductive technology
FSH: Follicle-stimulating hormone
E2: Estradiol
AFC: Antral follicle count
ICSI: Intracytoplasmic sperm injection
GV: Governor vessel
CV: Conception vessel
ST: Stomach
SP: Spleen
PC: Pericardium
TCM: Traditional Chinese medicine
BL: Bladder
KI: Kidney
HCG: Human chorionic gonadotropin
ROS: Reactive oxygen species
SOD: Superoxide dismutase
CRH: Corticotrophin-releasing hormone
GABA: Beta-aminobutyric acid
BAI: Baker anxiety self-rating scale
BDI-II: Baker depression self-rating scale
Zung-SAS: Zung anxiety self-rating scale
Zung-SDS: Zung depression self-rating scale
QOL: Quality of life measurement
CHQOL: Chinese quality of life scale
AEs: Adverse events
eCRF: Electronic case report form

## Declarations

### Dissemination policy

Findings will be published in peer-reviewed journals and presented at local, national, and international meetings and conferences to promote and explain the research to clinicians, patients, commissioners, and general medical community. We use the Clinical Trial Management Public Platform to manage and share our data. The data access is only for research, from January 2026 for 1 year.
