# Supplementary material for: Effect of electro-acupuncture on ovarian function of women with diminished ovarian reserve: study protocol for a randomized controlled trial
Source: Trials. 2021 Dec 14;22:921. doi: 10.1186/s13063-021-05894-2 (PMC8670117; doi:10.1186/s13063-021-05894-2)
Supplement: Supplementary file 2 — Additional file 2:. Informed consent [file 13063_2021_5894_MOESM2_ESM.docx]

**Informed consent · Information page**

Dear patient:

Your doctor has confirmed you as a diminished ovarian reserve(DOR) patient. We cordially invite you to participate in the study. This research program has been approved by the medical ethics committee of Tongji Hospital affiliated to Tongji Medical College of Huazhong University of Science and Technology, and is permitted to do clinical research.

Before you decide whether to participate in this study, please read the following carefully. This can help you understand the study and why it was conducted, the procedures and duration of the study, the benefits, risks, and discomfort it may bring to you. If you would like to, you could also discuss it with your relatives or friends, or ask your doctor for an explanation to help you make a decision.

**1. The background and purpose of the study**

**1.1 The burden of disease and present status of treatment**

Diminished ovarian reserve(DOR) is a common disease that leads to female infertility. With the rapid pace of today's society and the increasing of mental pressure and environmental pollution, the incidence rate of diminished ovarian reserve rises gradually, and is getting younger. There is no effective treatment with drugs at present.Acupuncture，with more than 3000 years history, is the main part of traditional Chinese medicine. In recent years, acupuncture, as a supplementary means treating gynecological and obstetric diseases, has drawn the worldwide attention. Acupuncture has already achieved satisfying results on treating gynecological diseases such as premature ovarian failure, polycystic ovarian syndrome, dysmenorrhea, pelvic inflammation, etc. The underlying mechanisms of the effect of acupuncture in the treatment of gynecological diseases may be via modulation of endogenous regulatory systems, including the sympathetic nervous system, the neuroendocrine system, the reproductive endocrinology system and the metabolic and endocrine system, and then effects the ovarian reserve function.

**1.2 Research objectives**

The aim of the study is to evaluate the effect of electro-acupuncture on ovarian function and following outcome of IVF-ET of diminished ovarian reserve(DOR) patients, and to evaluate its safety.

**1.3 Research departments and number of expected subjects**

We will include 338 DOR patients who take a visit to the departments mentioned below: Reproductive medicine center and Integrated traditional Chinese and western medicine department of Tongji Hospital attached by Tongji Medical College of Huazhong University of Science and Technology, Reproductive medicine center of Union Hospital attached by Tongji Medical College of Huazhong University of Science and Technology, Reproductive medicine center of Wuhan First Hospital, Reproductive Hospital affiliated to Jiangxi University of Traditional Chinese Medicine, Edwatz medical research institute. The age of the patients is between 18 to 40 years. According to the Network version of the central randomization system, patients are allocated randomly as 1:1 to treatment group and control group. Each group has 169 patients.

You will be included in this study if you are in the following condition:

1) Patients less than 40 years old, will do IVF-ET;

2) Low ovarian reserve: AFC<7, or AMH<1.1ng/ml; or has a history of poor ovarian response: in the last hyper stimulation cycle, the number of retrieved oocytes<3;

3) Spouse’ semen examination is normal, or after semen prewash can reach the standard of common IVF or Intracytoplasmic Sperm Injection(ICSI).

**2. Who should not participate in the study**

**You will not be included in this study if you are in the following condition:**

1) Male with azoospermia;

2) Male/female’s chromosome is abnormal;

3) Adenomyosis, uterine fibroids, endometrial polyps, scar uterine, reproductive system tuberculosis, oviduct effusion, pelvic lesions such as ovarian endometriosis cyst or tumor;

4) Female has other endocrine disease: thyroid diseases, hyperprolactinemia, insulin resistance, diabetes, adrenal diseases, etc.

5) Definitively diagnosed autoimmune diseases such as systemic lupus erythematosus, rheumatoid arthritis, antiphospholipid syndrome;

6) Other pathogenesis that leads to recurrent miscarriage or agnogenic recurrent miscarriage;

7) A history of cancer and has received radiotherapy and chemotherapy;

8) Had acupuncture treatment in recent 3 months;

9) Unwilling to sign the informed consent of this study.

In addition: ①Patients participating in other clinical study; ②Patients with other reasons that considered by investigators as inappropriate.

**What should I do if I participate in the study?**

**3.1** Before you are included in the study, your doctor will ask for and record your medical history, and conduct ultrasound and related hormone examination.

If you are an eligible subject, you can volunteer to participate in the study and sign the informed consent;

If you wouldn’t like to participate in the study, we will treat you as you wish.

**3.2 If you volunteer to participate in the study, the procedures will be conducted as below:**

You will be allocated to one of the two groups randomly: Treatment group (Acupuncture protocol 1) or Control group (Acupuncture protocol 2).

If you fulfil the inclusion criteria and accept our study design, baseline measurements will be done, including measurements of body weight, height, waist circumference, abdominal circumference, and fasting blood will be drawn for analyses of specific hormones. You will fill in questionnaires regarding of life quality related to health, symptoms of anxiety and depression, and sleep. When the bleeding which comes with spontaneous menstrual or the use of progesterone is finished, you should receive the acupuncture treatment in Integrated traditional Chinese and western medicine department of Tongji Hospital and Acupuncture department of Wuhan First Hospital. The acupuncture treatment is twice or three times a week during 2-3 months, in total 24 treatments. Treatments will be canceled during menstrual.

After the acupuncture treatment, we will evaluate your ovarian reserve function again, and then conduct the IVF-ET cycle treatment, this part of work will be according to your situation and conducted by the reproductive medicine center according to common procedure.

**3.3 Other items that need your cooperation**

You must come to the hospital at time as appointed. During the follow-up phase, your doctor may care about your condition by phone, WeChat, QQ or visit your home. Your follow-up is very important because your doctor will determine whether your treatment really works and guide you in time.

During the study, you cannot use other drugs that treat DOR or may influence ovarian function. When you need additional treatment, please contact your doctor in advance.

**4. Probable benefits of participating in the study**

Although there is evidence that acupuncture or electro-acupuncture works well for DOR, it is not guaranteed that it works for you. The electro-acupuncture used in this study is also not the only way to treat DOR. If it doesn't work for you, you can ask your doctor about possible alternative treatments. You will get sufficient treatment and doctors’ warmly care during the whole treatments. Hormone analyses and questionnaires will give you detailed information about your health status.

**5. Possible adverse reactions, risks and discomfort, inconveniences of participating in the study**

We will exclude subjects with a history of serious disease. We will record whether there are abnormal side effects. We will systematically collect the data of adverse reactions and severe adverse reactions. All the data will be quarterly reviewed by the data security oversight board, and the serious adverse reactions will be immediately ruled out. Personal information leakage may occur during the study. We will discuss about secrecy and privacy protection in details below.

The main risk is the side-effects of acupuncture. The main side-effects of acupuncture are local skin irritation, discomfort and vagal reaction (such as nausea and dizzy) during the operation. These abnormal conditions can be avoided by fully explaining how to eliminate patients' nervousness and concerns, choosing a comfortable and lasting patient position, skillful needle insertion and needle insertion, and strictly mastering acupuncture precautions. We will use a disposable treatment needle to avoid cross infection.

During the study period, if you have any discomfort, any new changes in your condition, or any unexpected circumstances, whether related to the study or not, you should inform your doctor in time and he/she will make a judgment and give appropriate medical treatment.

You need to go to the hospital for treatment on time during the study, and you need to do some examinations before and after the treatment, which will take up some of your time and may cause trouble or inconvenience to you.

**6. The costs**

The 2-3 months’ acupuncture treatments are free. In addition, you will receive the routine examination and treatment procedures for infertility, and the payment is on your own. The routine examination and treatment procedures are consistent in all our study centers. Before you agree to participate in this study, we will present and explain all the routine examination and treatment procedures. If you are lucky to be pregnant after treatments, you should pay for the cost of pregnancy and delivery.

Doctors will do their best preventing and treating possible injuries caused by the study. If adverse events occur in clinical trials, the committee of medical experts will determine if is related to acupuncture. The sponsor will provide the cost of treatment and the corresponding economic compensation for test-related damages in accordance with the provisions of China's code for the quality management of clinical trials for drugs.

Treatment and testing for other combined diseases will not be free of charge.

**7. Confidentiality for personal information**

Your medical records (research medical records /CRF, laboratory tests, etc.) will be kept intact in your hospital. The doctor will record the results of the tests and other examinations on your medical record. Researchers, ethics committees and drug regulatory authorities will be allowed to access your medical records. Your personal identity will not be disclosed in any public report on the results of this study. We will make every effort to protect the privacy of your personal medical data as far as the law allows.

According to medical research ethics, in addition to personal privacy information, test data will be available for public inquiry and sharing, and inquiry and sharing will be limited to web-based electronic databases to ensure that no personal privacy information will be disclosed.

**8. How to get more information?**

You can ask any questions about this study at any time and get answers accordingly.

Your doctor will keep you informed of any important new information that may affect your willingness to continue the study.

**9. It’s free participating in or dropping out of the study**

Whether you participate in the study entirely depends on you. You may refuse to participate in this study or withdraw from this study at any time during the study, which will not affect your relationship with your doctor, nor will it affect the loss of your medical care or other benefits.

For the best interest of you, a doctor or researcher may discontinue your participation at any time during the study.

If you withdraw from the study for any reason, you may be asked about your use of acupuncture. You may also be required to have a laboratory and physical examination if the doctor deems it necessary.

**10. What should I do now?**

It depends on you (and your family) to participate in this study.

Please ask your doctor as many questions as possible before you make your decision to participate in the study.

Thank you for reading the above materials. If you decide to participate in this study, please tell your doctor that he or she will arrange for you all matters related to the study. Please keep this information.

**Informed consent · Signature page**

**The name of the clinical study project: Effect of electro-acupuncture on ovarian function and outcome of IVF-ET of women with diminished ovarian reserve(DOR): A Randomized controlled trial.**

**Project undertaking department:** **Tongji Hospital attached by Tongji Medical College affiliated to Huazhong University of Science and Technology.**

**Project cooperating department:** **Union Hospital attached by Tongji Medical College affiliated to Huazhong University of Science and Technology, Wuhan First Hospital, Reproductive Hospital affiliated to Jiangxi University of Traditional Chinese Medicine, Edwatz medical research institute.**

**Project assignment number: AE-DOR (Number: 3.0)**

**Declaration of Consent**

I have read the above introduction of the study and have the opportunity to discuss this study with my doctor and ask questions. All my questions have been answered satisfactorily.

I know the risks and benefits of participating in this study. I know that participating in the study is voluntary, and I am sure that there is sufficient time to consider it, and I understand that:

I can ask my doctor for more information at any time.

I can withdraw from this study at any time without being discriminated or reprimanded, and my medical treatment and rights and interests will not be affected.

I am also aware that if I withdraw from the study at the middle of the study, especially when I withdraw from the study due to drug reasons, it will be very beneficial for the whole study if I tell the doctor about my changes of illness and complete the corresponding physical examination and physical and chemical examination.

If I need to take any other medication because of the change in my condition, I will consult my doctor in advance or tell the doctor truthfully afterwards.

I agree that the ethics committee of the drug regulatory authority or the representative of the applicant should consult my research materials.

I will receive a signed and dated copy of the informed consent.

In the end, I decide to agree to participate in this study, and I promised to follow the doctor's instructions.

Patient’s signature: Date:

Phone:

I confirm that I have explained the details of the trial to the patient, including its rights and potential benefits and risks, and have given a signed copy of the informed consent.

Doctor’s signature: Date:

Doctor’s work phone:
